# Claudin-19 Is Regulated by Extracellular Osmolality in Rat Kidney Inner Medullary Collecting Duct Cells

**DOI:** 10.3390/ijms20184401

**Published:** 2019-09-07

**Authors:** Annalisa Ziemens, Svenja R. Sonntag, Vera C. Wulfmeyer, Bayram Edemir, Markus Bleich, Nina Himmerkus

**Affiliations:** 1 Institute of Physiology, Christian-Albrecht-University Kiel, Hermann-Rodewald-Str. 5, 24118 Kiel, Germany; ziemens.annalisa@gmail.com (A.Z.); Svenja.Sonntag@web.de (S.R.S.); wulfmeyer.vera@mh-hannover.de (V.C.W.); m.bleich@physiologie.uni-kiel.de (M.B.); 2 Department of Ophthalmology, University of Lübeck, Ratzeburger Allee 160, 23538 Lübeck, Germany; 3 Department of Nephrology and Hypertension, Hannover Medical School, Carl-Neuberg-Str. 1, 30625 Hannover, Germany; 4 Department of Internal Medicine IV, Hematology and Oncology, University Hospital Halle, Ernst-Grube-Str. 40, 06120 Halle (Saale), Germany; bayram.edemir@uk-halle.de

**Keywords:** barrier function, paracellular permeability, antidiuretic hormone

## Abstract

The inner medullary collecting duct (IMCD) is subject to severe changes in ambient osmolality and must either allow water transport or be able to seal the lumen against a very high osmotic pressure. We postulate that the tight junction protein claudin-19 is expressed in IMCD and that it takes part in epithelial adaptation to changing osmolality at different functional states. Presence of claudin-19 in rat IMCD was investigated by Western blotting and immunofluorescence. Primary cell culture of rat IMCD cells on permeable filter supports was performed under different osmotic culture conditions and after stimulation by antidiuretic hormone (AVP). Electrogenic transepithelial transport properties were measured in Ussing chambers. IMCD cells cultivated at 300 mosm/kg showed high transepithelial resistance, a cation selective paracellular pathway and claudin-19 was mainly located in the tight junction. Treatment by AVP increased cation selectivity but did not alter transepithelial resistance or claudin-19 subcellular localization. In contrast, IMCD cells cultivated at 900 mosm/kg had low transepithelial resistance, anion selectivity, and claudin-19 was relocated from the tight junctions to intracellular vesicles. The data shows osmolality-dependent transformation of IMCD epithelium from tight and sodium-transporting to leaky, with claudin-19 expression in the tight junction associated to tightness and cation selectivity under low osmolality.

## 1. Introduction

The inner medullary collecting duct (IMCD) is the last segment of the nephron. It is embedded in the unique structure of the inner medulla together with descending and ascending thin limbs of Henle’s loop and the accompanying vasa recta, which are specialized capillaries [1,2]. Maintenance of body water homeostasis under extreme variations in water intake is achieved under hormonal regulation, especially AVP signaling. Thereby, urine osmolality can vary between 50 mosm/kg and 1500 mosm/kg in humans. Of high importance is the interstitial osmolality provided by the main osmolytes, NaCl and urea, and the cortico-medullary concentration gradient established by the loop of Henle [3,4] countercurrent concentration system. During antidiuresis, interstitial and intraluminal osmolality is high and the transepithelial gradient is levelled by the water flux from the lumen to the interstitium through water channel insertion into the cell membrane. Under water diuresis, interstitial osmolality is lower; however, high NaCl concentration is conserved and mainly urea concentration is changing with diuretic state [5,6]. Therefore, the now transcellularly water tight collecting duct faces a high transepithelial osmotic gradient. The interstitial accumulation of urea during antidiuresis is tightly controlled by antidiuretic hormone (AVP) but also by other factors (e.g., endothelin, PGE2) [4]. Hypertonicity and urea are thereby cell stressors and cellular osmo-homeostasis has to be controlled and adapted under these changing conditions [7,8]. Osmo-protective responses include accumulation of compatible organic osmolytes, abundance of heat shock proteins, and control of transmembrane water flux by water channels. Ionic and osmotic gradients under both antidiuresis or water diuresis between the luminal and the interstitial side of the epithelium do not only challenge the transcellular barrier, but also the paracellular route. The paracellular cleft between epithelial cells is specifically sealed by the tight junction (TJ). This complex is composed of a variety of different proteins fulfilling a broad spectrum of tasks in addition to barrier formation and provision of permeability, including anchoring the TJ proteins to the cytoskeleton and cell signaling. The proteins responsible for tightness and permeability are claudins, a family comprising more than 25 different members to date (for review, see [9,10,11]). From a functional perspective, IMCD paracellular properties can vary with respect to water permeability and ion selectivity. Under diuretic conditions, in the absence of AVP, the collecting duct is sealed against water permeation [12]. In antidiuresis, water channels provide a high transcellular water permeability. Early studies in the 1990s by Flamion et al. suggested a potential additional paracellular route for water in IMCD under strong antidiuresis [13]. So far, different claudins have been described for collecting ducts (claudin-3, -4, -7, -8, -10, -19) [14]. Especially, claudin-4 and -8 have been implicated in paracellular selectivity and ion transport [15]. Claudin-19 was described first in 2004 [16] and in the kidney, it is mainly expressed in the thick ascending limb of Henle’s Loop (TAL) [17,18,19]. Mutations in humans lead to familial hypomagnesemia with hypercalciuria and nephrocalcinosis (FHHNC, OMIM 610036, [17]) which also can be caused by mutations in claudin-16 [20,21]. Both TAL claudins are required for functional TAL Ca^2+^ and Mg^2+^ transport [19,22]. In 2006, Lee et al. described a different expression pattern identified by antibody staining, showing claudin-19 in the distal tubule (i.e., mainly TAL) and the collecting duct in humans and rats [23]. In RNA-seq analysis of microdissected rat kidney tubule segments, claudin-19 mRNA has been found in more upstream collecting duct segments but hardly any in IMCD [24]. By immunofluorescence staining, our group repetitively encountered claudin-19 expression in segments more distal to the TAL and decided to investigate its expression and function in the inner medulla.

We hypothesize that claudin-19 is part of the IMCD TJ, that IMCD cells adapt to osmolality or AVP stimulation, and that claudin-19 takes part in this adaptation. We used the well-established model of primary cultivated inner medullary collecting duct cells grown on permeable supports (IMCDs) [25,26]. Cells were cultivated under hormone stimulation and different osmotic challenges. Under cell culture conditions with proliferating cells not yet confluent and therefore not yet forming a complete epithelial layer, naturally occurring highly variable osmotic conditions are not easy to copy. We focused on long term (days) basolateral changes, omitting transepithelial gradients. Plasma-isotonicity represented cortical conditions and two increasing medullary osmolalities represented either the medullary axis or different diuretic states. Under low osmolality, claudin-19 localized to the TJ of IMCD, most likely fulfilling the sealing function and being responsible for high transepithelial resistance.

## 2. Results

### 2.1. Claudin19 Is Expressed in Inner Medullary Collecting Duct

**Figure 1:**
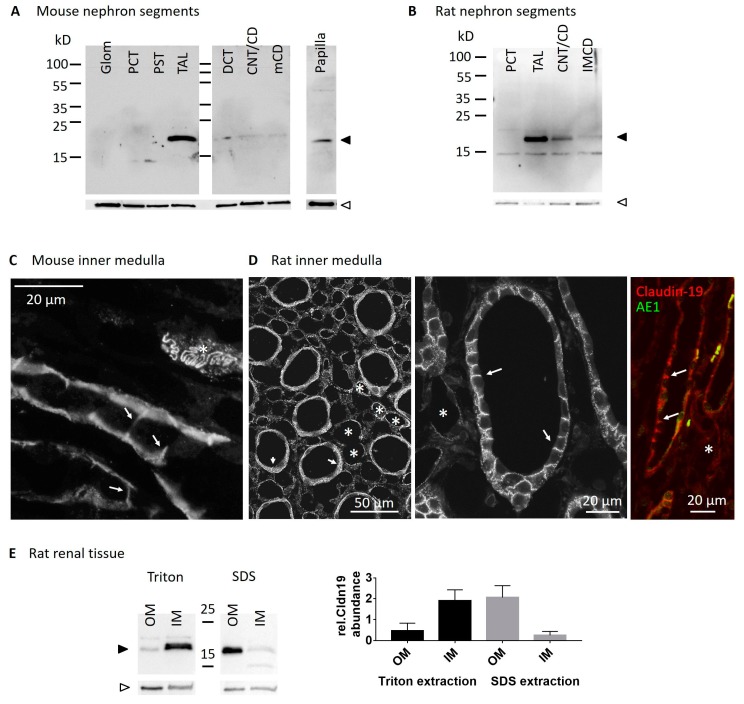
Claudin-19 in rodent kidney tissue. (**A**) Western blot of mouse kidney segments: glomeruli (Glom), proximal convoluted tubule (PCT), proximal straight tubule (PST), thick ascending limb (TAL), distal convoluted tubule (DCT), connecting tubule/cortical CD (CNT/CD), medullary CD (mCD), and papilla. Upper part: claudin-19 (black arrowhead), lower part: β-actin (white arrowhead). (**B**) Western blot of rat kidney segments: proximal convoluted tubule (PCT), thick ascending limb (TAL), connecting tubule and cortical CD (CNT/CD) and inner medullary collecting duct (IMCD). Claudin-19 and β-actin indicated as in A. Immunofluorescence of claudin-19 in mouse (**C**) and rat (**D**) inner medulla, arrows indicate claudin-19 TJ expression in IMCD, asterisks indicate thin limbs. AE1 (green) costaining in claudin-19 (red) positive IMCD in rat inner medulla. (**E**) Western blot of rat kidney tissue: Triton and SDS solubilized protein extracts of Cortex, outer medulla (OM) and inner medulla (IM), claudin-19 and β-actin indicated as in A. Summary of *n* = 2 independent extraction experiments showing Triton solubilization mainly in IM and SDS solubilization mainly in OM.

We investigated claudin-19 expression using WB analysis in both mouse and rat tissue. Along the nephron of the mouse (Figure 1A), claudin-19 protein was absent in glomeruli and proximal tubule (PT). In TAL and all segments further downstream, claudin-19 was expressed and protein abundance was highest in TAL, medullary CD (mCD), and in papilla tissue. Qualitatively similar results were obtained along the rat nephron and here we could specifically detect claudin-19 also in dissected and isolated IMCD (Figure 1B). We performed claudin-19 immunofluorescence staining in mouse and rat inner medulla (Figure 1C,D). Claudin-19 was expressed in both, IMCD and thin limbs. In IMCD, claudin-19 was located mostly in the TJ, i.e., line- or dot-shaped fluorescence along the apical cell cleft but also extending to the lateral cell borders (Figure 1D). In addition, some intracellular vesicular staining was visible at the basal cell pole of the IMCD cells. Costaining with the intercalated cell anion exchanger AE1 confirmed IMCD localization. Thin limb TJs showed the typical meandering pattern (Figure 1C [27]). Next, we investigated claudin-19 protein in rat kidney tissue in a more quantitative approach and tried to extract membrane proteins first using the mild nonionic detergent triton-x-100 (Triton), followed by a harsher extraction of the remaining membrane proteins by incubation with the anionic surfactant sodium dodecyl sulfate (SDS). As shown in Figure 1E, inner medullary claudin-19 is mainly already solubilized by Triton extraction, whereas outer medullary claudin-19 (i.e., mainly TAL) only solubilized in the presence of SDS, indicating a different TJ structure and organization.

### 2.2. AVP Signaling Is Preserved in IMCD Cells at Different Osmotic Culture Conditions

**Figure 2:**
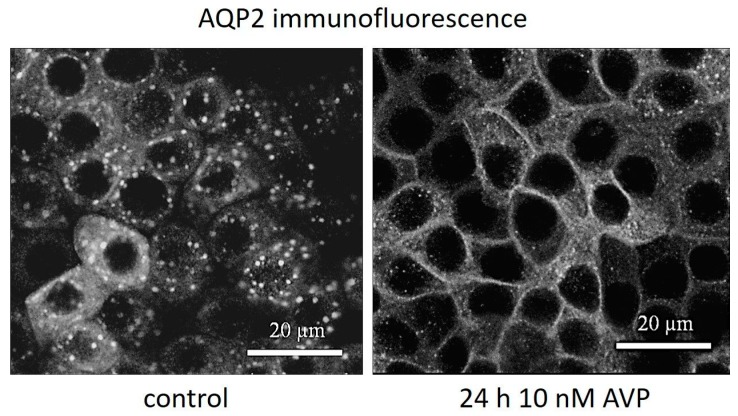
Effect of antidiuretic hormone (AVP) on subcellular AQP2 localization in 300-IMCD. AVP stimulation induced AQP2 insertion into the plasma membrane.

In a first set of experiments, we tested the IMCD culture cells for basic physiological function. Therefore, IMCD cells were cultured under 300 mosm/kg (300-IMCD) and 600 mosm/kg (600-IMCD) conditions for five days and finally treated for 24 h with AVP. As shown in Figure 2 for 300-IMCD, subcellular localization of AQP2 was assessed by immunostaining. Although some AQP2 still remained in intracellular vesicles, AQP2 membrane staining markedly increased, revealing intact AVP signaling also after several days of cell culture. The same results were obtained for 600-IMCD (data not shown).

### 2.3. AVP Treatment Changes IMCD Cell Electrophysiological Properties but not Claudin-19 Localization

**Figure 3:**
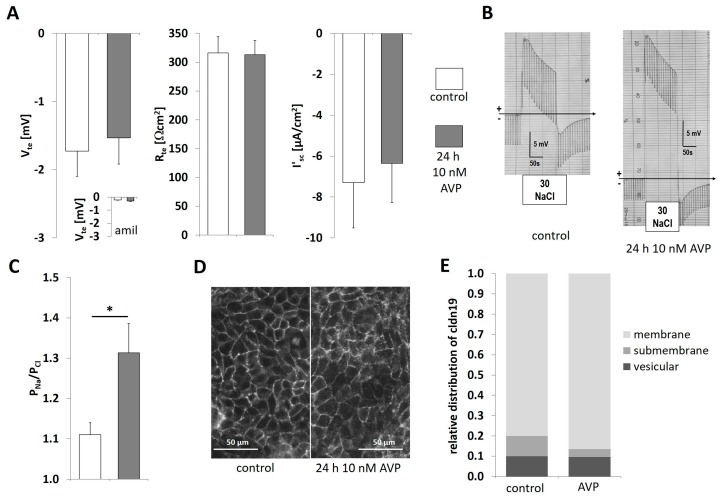
300-IMCD under AVP stimulation (10 nmol/L). (**A**) Electrophysiological properties with transepithelial voltage V_te_, transepithelial resistance R_te_, and equivalent short-circuit current I’_sc_, with or without 24 h prestimulation with AVP. Insert in V_te_ panel: V_te_ in the presence of 50 µM amiloride. (**B**) Original experiments showing 30 mmol/L NaCl diffusion potentials, (**C**) summarized permeability ratio PNa/PCl. Data are means ± SEM, *n* = 13,13; * *p* < 0.05. (**D**) Immunofluorescence of claudin-19 in 300-IMCD with or without 24 h prestimulation with AVP, (**E**) summarized subcellular localization of claudin-19 in the categories of membrane TJ staining, submembrane and intracellular vesicular staining, *n* = 13, 13.

**Figure 4:**
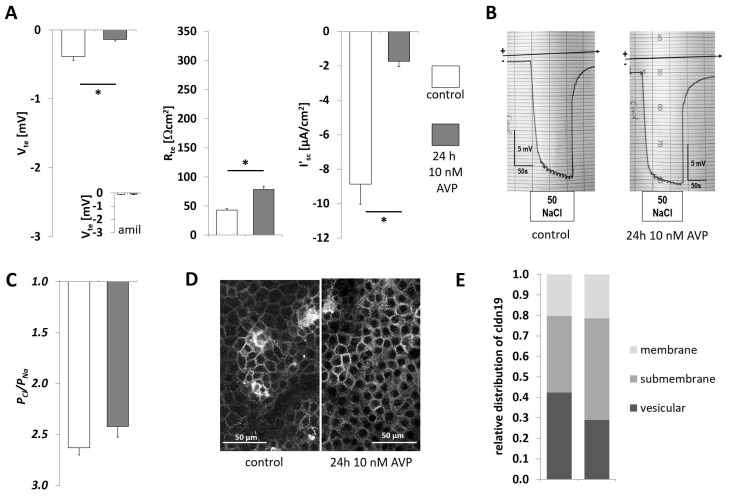
600-IMCD under AVP stimulation (10 nmol/L). (**A**) Electrophysiological properties with transepithelial voltage V_te_, transepithelial resistance R_te_, and equivalent short-circuit current I’_sc_, with or without 24 h prestimulation with AVP. Insert in V_te_ panel: V_te_ in the presence of 50 µM amiloride. (**B**) Original experiments showing 50 mmol/L NaCl diffusion potentials, (**C**) summarized permeability ratio P_Cl_/P_Na_. Data are means ± SEM, *n* = 12,12; * *p* < 0.05. (**D**) Immunofluorescence of claudin-19 in 600-IMCD, (**E**) summarized subcellular localization of claudin-19 in the categories of membrane TJ staining, submembrane and intracellular vesicular staining, *n* = 8, 7.

In two independent experimental series, 300-IMCD (Figure 3) or 600-IMCD (Figure 4) were cultivated for 24 h in the absence (control) or presence of 10 nmol/L AVP prior to the electrophysiological measurements. Under 300 mosm/kg conditions, mimicking strong water-diuresis or cortical osmolality, IMCD cells showed low, but consistently negative transepithelial voltage (V_te_) and a transepithelial resistance (R_te_) in the range of 310 Ωcm^2^. These properties were not changed by AVP treatment. Accordingly, equivalent short-circuit current I’_sc_ also remained unaltered by AVP treatment (Figure 3A). Paracellular permeability properties were tested in the continuous presence of 50 µmol/L amiloride (V_te_ in the presence of amiloride was virtually abolished as shown in insert in Figure 3A) by applying an iso-osmotic NaCl concentration gradient with low NaCl (30 mmol/L) at the luminal side. In Figure 3B, two representative original chart recordings are shown to illustrate the development of a lumen-positive diffusion potential (DP) after application of the iso-osmotic NaCl gradient, indicating preferred diffusion of Na^+^ ions towards the lumen. After AVP treatment, DP increased, resulting in higher cation selectivity as summarized in Figure 3C. To test whether claudin-19 subcellular localization was altered by AVP stimulation, we compared filters of both groups for the relative distribution of claudin-19 within the cells. Under control as well as under AVP treatment, almost all claudin-19 staining was localized in thin lines representing membrane, i.e., TJ staining (Figure 3D,E). There was no difference in the tight junction scores (2.70 ± 0.06 and 2.77 ± 0.05), respectively.

The same parameters were also measured under 600 mosm/kg (Figure 4) culture conditions, which is a more physiological osmolar condition for inner medulla. Osmolality was adjusted by adding urea as well as NaCl. In comparison to 300-IMCD (Figure 3), V_te_ and R_te_ of 600-IMCDs was lower. In addition, V_te_ and I’_sc_ became less negative under AVP treatment, whereas R_te_ increased (Figure 4A). DP was measured after luminal exchange to iso-osmotic 50 mmol/L NaCl to generate a NaCl concentration gradient comparable to the previous experiments in 300-IMCD. In absolute contrast to 300-IMCD (Figure 3B,C), we now observed lumen negative diffusion voltage (Figure 4B), indicating pronounced anion selectivity (Figure 4C). There was no significant effect of AVP on paracellular selectivity as shown in original recordings (Figure 4B) and summary (Figure 4C). Claudin-19 subcellular distribution was not different between control situation und AVP treatment (Figure 4D,E; tight junction score 1.76 ± 0.07 and 1.93 ± 0.03, respectively). However, claudin-19 subcellular distribution was again markedly changed by osmolality, with a shift to submembrane and even more intracellular vesicular representation at 600-IMCD.

### 2.4. Differences in Extracellular Osmolality Determine IMCD-Cell Trans- and Paracellular Properties

**Figure 5:**
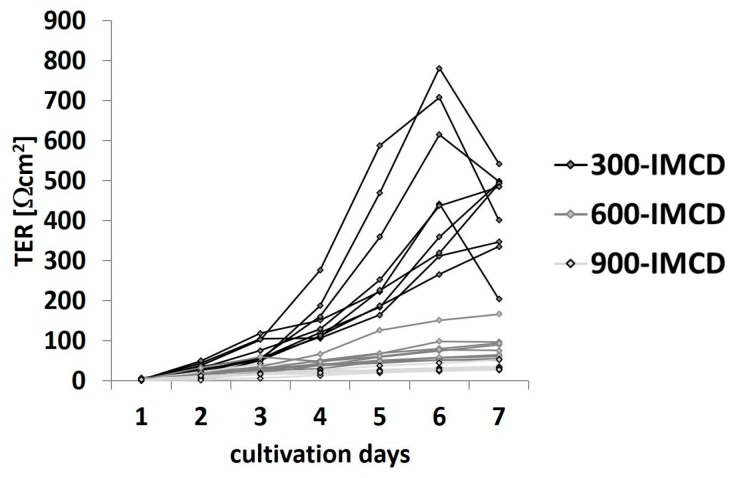
Time course of transepithelial resistance (TER) under culture conditions after seeding for nine different preparations with the filters of 300-IMCD, 600-IMC, and 900-IMCD, respectively.

The observation that osmolality strongly changed the transepithelial properties of IMCD-cells prompted us to further examine this strikingly strong effect of ambient osmolality on claudin-19 function. Therefore, we performed a new independent set of experiments cultivating IMCD cells in parallel under 300 mosm/kg, 600 mosm/kg, and 900 mosm/kg (900-IMCD) to mimic stronger antidiuresis. Transepithelial resistance measured daily under culture condition (TER) was monitored and cells reached peak resistance values at day 6 under all three osmolality regimens (Figure 5). Interestingly, the average resistance varied considerably between the three groups. Whereas cells grown under plasma isotonic conditions (300-IMCD) reached TER values of around 550 Ωcm^2^, TER of 600-IMCDs was five times and TER of 900-IMCDs even 10 times lower.

**Figure 6:**
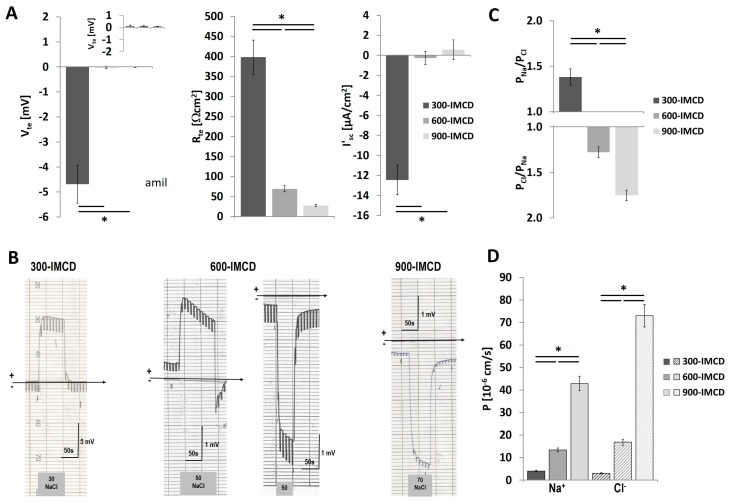
Comparison between 300-IMCD, 600-IMCD, and 900-IMCD after five days of cultivation. (**A**) Electrophysiological properties with transepithelial voltage V_te_, transepithelial resistance R_te_ and equivalent short-circuit current I’_sc_. Insert in Vte panel: Vte in the presence of 50 µM amiloride. (**B**) Original experiments showing 30 mmol/L NaCl (300-IMCD), 50 mmol/L (600-IMCD), and 70 mmol/L (900-IMCD) diffusion potentials. (**C**) Summarized permeability ratio PNa/PCl and PCl/PNa, respectively. (**D**) Calculated Na^+^ and Cl^-^ permeabilities. Data are means ± SEM, *n* = 24, 39, 46; * *p* < 0.05.

Subsequently, filters were measured in Ussing Chamber experiments at day 5. Figure 6 shows electrophysiological properties in more detail. Only 300-IMCDs showed noteworthy lumen negative V_te_, which disappeared under high osmolality. The resistance measurements during cell culture (TER, Figure 5) were corroborated. 300-IMCD displayed ca. 400 Ωcm^2^, 600-IMCDs ca. 70 Ωcm^2^, and 900-IMCD only ca. 27 Ωcm^2^ (Figure 6A). Consistently, only 300-IMCD showed a lumen negative transcellular transport current I’_sc_ in this set of experiments. Paracellular selectivity was then assessed using comparable transepithelial NaCl concentration gradients adjusted to medium NaCl concentrations, respectively (Table 1, Figure 6B). Whereas 300-IMCD consistently showed a lumen positive DP, indicating cation selectivity (left original recording in Figure 6B), and all 900-IMCD a lumen negative DP, indicating anion selectivity (right original recording in Figure 6B), 600-IMCD displayed either lumen positive or lumen negative DP. Ion permeability ratios for Na^+^ and Cl^−^ are summarized in Figure 6C. 300-IMCDs were cation selective and 900-IMCDs anion selective, 600-IMCD showing a moderate anion selectivity close to unity. Absolute permeability was calculated from these ratios and R_te_. As the effect of osmolality on R_te_ of IMCD was much more pronounced than the changes in ion selectivity, the effect of higher osmolality was an increase for P_Na_ and even more for P_Cl_. Figure 6D depicts the calculated permeabilities for Na^+^ and Cl^−^ with an up to 25-fold increase in P_Cl_ comparing 300-IMCD and 900-IMCD.

### 2.5. Effect of the Different Osmolality on IMCD Transport Properties

**Figure 7:**
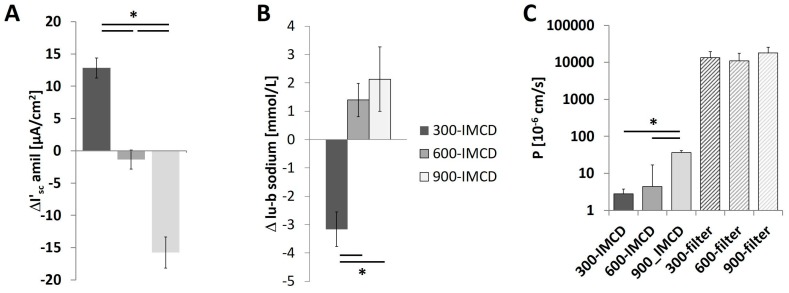
Transepithelial transport parameters of 300-IMCD, 600-IMCD, and 900-IMCD after five days’ cultivation. (**A**) Amiloride-sensitive equivalent short-circuit current ΔI’_sc_. (**B**) Na^+^ concentration difference between luminal and basolateral cultivation medium, (**C**) summarized 40 kD FITC–dextran permeability. Data are means ± SEM, IMCD filters: *n* = 10,11,11; empty filters: *n* = 3, 3, 3; * *p* < 0.05.

300-IMCD showed a lumen negative I’_sc_ which was almost completely abolished by luminal amiloride application, indicating functionally expressed ENaC (Figure 7A). 600-IMCD showed basically no amiloride-dependent I’_sc_ but, astonishingly, 900-IMCD became slightly lumen positive after amiloride application. Sodium transport was verified by the measurement of changes in sodium concentration under cell culture conditions within 24 h. Luminal and basolateral cell culture medium was collected and compared directly before the Ussing chamber experiments. Only 300-IMCD showed sodium reabsorption decreasing the luminal sodium concentration (Figure 7B). 600-IMCD and 900-IMCD did not achieve a significant difference in luminal versus basolateral sodium concentration. To investigate whether osmolality affected the overall tightness of the epithelium for larger molecules, we applied 40 kDa FITC–dextran to one compartment of the cell culture and measured the fluorescence in the trans-compartment. In comparison to the empty cell culture filters, confluent cell layers showed hardly any FITC–dextran permeability confirming intact cell layers. Only 900-IMCD showed a slightly higher 40 kD FITC–dextran permeability (Figure 7C), still minute in comparison to empty filters.

### 2.6. Differences in Extracellular Osmolality Change Claudin-19 Subcellular Localization

**Figure 8:**
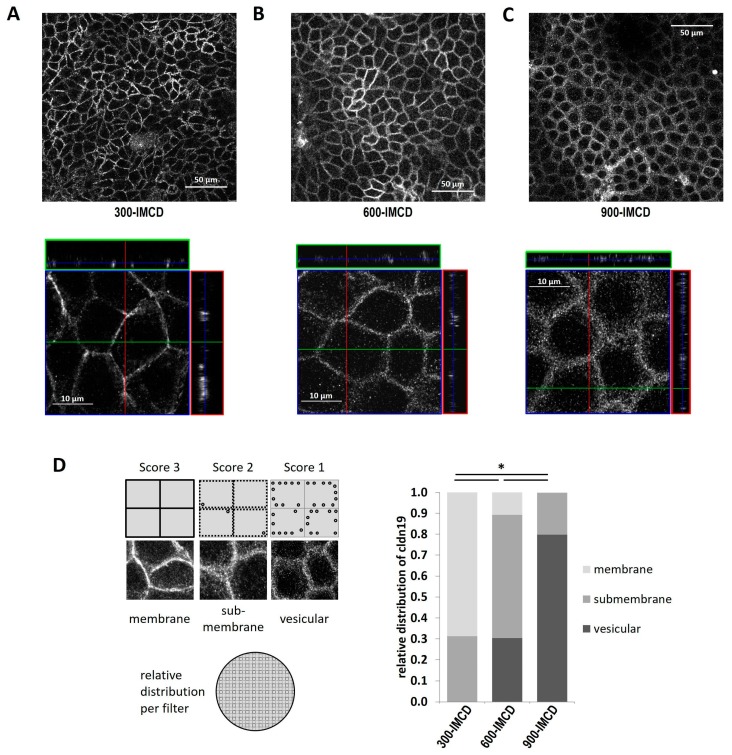
Immunofluorescence of claudin-19 (**A**) in 300-IMCD, (**B**) in 600-IMCD, and (**C**) in 900-IMCD, shown in an xy-overview (upper panel) and a representative z-stack (lower panel). (**D**) Summarized subcellular localization of claudin-19 in the categories of membrane TJ staining, submembrane and intracellular vesicular staining (schematic drawing), *n* = 12, 14, 12.

Finally, we compared the subcellular localization of claudin-19 in the different IMCD cultures and corroborated the findings already implicated by the AVP experiments. Whereas in 300-IMCD most claudin-19 was localized to the tight junction and appeared as a sharp line (Figure 8A), the percentage of submembrane and intracellular vesicular staining increased at higher cell culture medium osmolality (Figure 8B,C). Representative immunofluorescence stainings for claudin-19 are shown in (Figure 8A–C) and the observations are summarized as relative distribution between membrane, submembrane, and vesicular compartments (Figure 8D). 300-IMCD showed nearly 70% TJ staining (TJ score 2.69 ± 0.10), 600-IMCD expression pattern was intermediate (TJ score 1.80 ± 0.11), and in 900-IMCD, more than 90% of claudin-19 was distributed to small intracellular vesicular-like structures (TJ score 1.13 ± 0.08).

### 2.7. Further Observations

**Figure 9:**
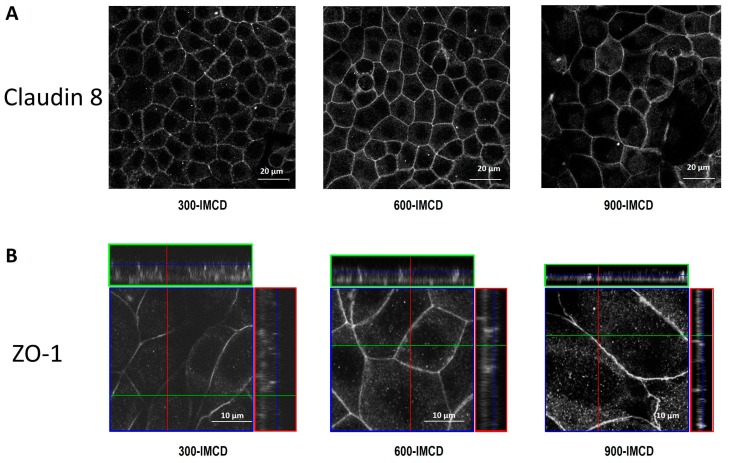
Immunofluorescence of claudin-8 in xy-overview in 300-IMCD, 600-IMCD, and 900-IMCD (**A**). Z-stacks of immunofluorescence of ZO-1 in 300-IMCD, 600-IMCD, and 900-IMCD (**B**).

This study mainly focused on the expression and localization of claudin-19 under different osmotic cell culture conditions. However, in comparison to the changing subcellular localization of claudin-19, which we investigated in detail, we could not find a similar pattern in the expression of claudin-8 (Figure 9A). In addition, the TJ associated scaffolding protein ZO-1 localized nicely to the tight junction belt of IMCD under all conditions (Figure 9B), independent of osmolality.

Furthermore, the z-stacks in Figure 8; Figure 9 show that osmotic cell culture conditions also influenced cell height and size. Especially 900-IMCD, although covering the total area of the filter, were rather flat and more expanded, suggesting a reduced number of available cells.

## 3. Discussion

We and others have investigated the expression and function of a variety of claudins along the nephron [28,29]; however, there are still several claudins in this large protein family which are not fully characterized with respect to their expression and functional role. Refinement of Western Blot analysis and immunofluorescence of claudin-19 protein in isolated renal tubules showed substantial expression in medullary and inner medullary collecting ducts. As these tubules are exposed to varying interstitial osmolality depending on the diuretic state and at the same time have to serve different needs with respect to permeability and barrier function, we hypothesized that claudin-19 is involved in medullary and inner medullary collecting duct function and might be under the control of antidiuretic hormone and osmolality.

We used primary IMCD cells cultivated under hormonal and osmotic challenges to investigate their electrophysiological transport properties as well as subcellular localization of claudin-19 under these conditions. AVP partially influenced electrophysiological properties and did this in an osmolality depended manner: 300 mosm/kg—increase in cation selectivity (Figure 3), 600 mosm/kg—decrease in transcellular transport and increase in R_te_ (Figure 4). Claudin-19 subcellular localization, however, remained unaffected by the hormone. More strikingly, low osmolality increased R_te_ substantially and translocated claudin-19 to the tight junction, whereas high osmolality resulted in rather low R_te_ and removal of claudin-19 from the TJ to intracellular vesicles (Figure 6, Figure 7 and Figure 8). In addition, paracellular selectivity changed from cation to anion selectivity with increasing extracellular osmolality.

Although the data are shown in comparison to isotonic conditions, we consider 600 mosm/kg as “physiological” osmolality for our experiments, a tonicity more attributed to the outer medulla [3] and the more outer parts of inner medulla. This is in line with former publications [25,30] and takes the cone-like structure of the inner medulla into account. However, towards the tip of the papilla, even higher osmolalities are to be expected in rodents as rats, which excrete urine with osmolality values between 1500 and 2500 mosm/kg [27,31]. The electrophysiological findings of our primary cell culture under hyperosmotic conditions (600-IMCD and 900-IMCD) reproduced the findings from native, isolated perfused papillary collecting ducts rather well, with a slightly lumen positive V_te_ (< 1 mV) and R_te_ of about 148 Ωcm^2^ [32]. We could corroborate the presence of claudin-19 protein in IMCD in native tissue (Figure 1) as well as in primary cell culture (Figure 3, Figure 4 and Figure 8). This is in accordance with the finding of Lee et al. [23] but did not find its way into the recently published expression profiles along the nephron where claudin-19 so far is not included in the collecting duct [28,29]. Our data is also in line with the transcriptome, which is negative for the tubule segments upstream the TAL but positive for the TAL and downstream, including IMCD. The lower metabolism of cells in the medulla might explain why protein abundance is high in comparison to the low mRNA levels found for claudin-19 in the IMCD transcriptome [24].

Acute effects of changes in osmolality on paracellular properties have been investigated mainly in established renal epithelia cell lines. In MDCK cells, mimicking proximal tubules, small steps of osmolality increase (10–20 mosm/kg by addition of glucose or mannitol) were tested to imitate diabetic nephropathy with conflicting results. After 24–72 h hyperosmotic challenge using glucose, R_te_ decreased, while cation selectivity increased (increase in claudin-2 expression and reduction in claudin-1 and -3; [33]). In contrast, mannitol increased R_te_ by decreasing claudin-2 expression and increasing claudin-1 within the same time-frame [34]. Collecting ducts face more pronounced changes in osmolality due to the unique urinary concentration mechanisms [4]. A lot of studies have been performed in IMCD3 cells, collecting duct cells originally derived from mouse inner medulla, challenging them acutely by increasing tonicity and looking for cellular adaptations within hours or the first days. Fast changes to higher osmolality triggered apoptotic events [35]; a combination of NaCl and urea was better tolerated [36]. Altogether, IMCD3 adapted well to higher osmolality if changes were induced stepwise [37]. In primary IMCD cells, changes in tonicity also lead to adaptation, partially to apoptosis and to vast changes in gene expression [8,26]. In our experiments, we observed tonicity-dependent changes in cell shape, with expanded and flat cells under high osmolality. This might be attributed to the higher susceptibility of the cells to undergo apoptosis. Still the epithelial monolayer was intact. In this study, we did not further investigate apoptosis, but we confined our experiments on physiological transport properties and tight junction protein expression. Adaptation described for IMCD3 also included changes in the TJ proteins [38,39]. Claudin-4 was thereby under the control of the scaffolding multi-PDZ domain protein 1 (MUPP-1) and showed higher expression in IMCD3 cells adapted to high osmolalities. In native murine papilla, claudin-4 expression was upregulated after water deprivation of mice [39]. In addition, we could show that ascending thin limbs reacted to water load by increasing the cation preference of their paracellular pathway [27]. Otherwise, not much is known about inner medullary adaptation of the paracellular pathway to changing environments. For this study, we focused our investigation mainly on claudin-19 in long-term adaptation experiments and on functional states.

Plasma isotonic conditions are most probably an environment rarely faced by IMCD cells in native inner medulla. One explanation for the changes seen here, especially functional ENaC expression (Figure 7A) and directed Na^+^ transport (Figure 7B), could be that the cells undergo a transition to cortical collecting duct properties; a kind of ‘corticalization’. All three ENaC subunits are expressed in all parts of the connecting tubule and the collecting duct [40]. However, without aldosterone stimulation, functional ENaC in the luminal membrane plays a crucial role mainly in the connecting tubule [41] and even in the cortical collecting duct of mice, lumen negative V_te_ as an indicator of the presence of ENaC is not measured [42] in the absence of aldosterone. As already mentioned, IMCDs did not show negative V_te_ [32]. Accordingly, water homeostasis has been implicated in the regulation of ENaC. Crambert et al. could show that water deprivation in mice increased ENaC expression in cortical, but not outer medullary CDs, and in a collecting cell line, hyperosmolality sufficed to reduce ENaC expression [43]. On the other side, low osmolality at the luminal side, but not necessarily at the basolateral side, would be indicative for water diuresis, which would require a sealing against water flux and the assimilation of luminal and basolateral osmolalities [12].

How could known claudin-19 properties support collecting duct function? Under isotonic conditions, claudin-19 appears to be localized in the TJ (Figure 3 and Figure 8). In TAL, claudin-19 confers Ca^2+^ and Mg^2+^ permeability to the tight junction, together with claudin-16 [22,44]. If claudin-19 alone is expressed in MDCK cells, it seems to act as cation barrier [18], whereas expression in a per-se anion selective background (porcine kidney cells, LLC-PK1), claudin-19 reduces anion selectivity [45]. Altogether, translocation of claudin-19 to the tight junction might be responsible for the increase in R_te_ and the accompanying cation selectivity. So far, no other direct interaction partners of claudin-19, in analogy to claudin-16/19 interaction, have been described. Obviously, the TJ structure and accessibility to detergents was remarkably different between the outer and the inner medulla (Figure 1). The TAL presents the main tubular component of the inner stripe of outer medulla (we excluded the outer stripe for a more secure exclusion of cortex), whereas in the inner medulla, the collecting duct is probably the main component by cell mass. Hence, outer medulla claudin-19 originates mainly from TAL and inner medulla claudin-19 mainly from IMCD, both showing completely different dissolving profiles. It has been proposed that the tight junction complex can be part of or be associated with cholesterol-enriched “lipid rafts” of plasma membranes. These lipid rafts consist of cholesterol and sphingomyelin to form a tightly packed membrane lipid region [46]. The extractability of, e.g., claudin-4, is strongly dependent on the detergent used for solubilization [47]. Triton solubility increases with lipid raft disruption. Sugibayashi et al. showed in MDCK cells that this disruption increased solubility to Triton for claudin-4, -5, and occludin, but not for claudin-1, -2, and -3, indicating that in this cell system, claudin-4 and claudin-5 were organized in lipid rafts, whereas claudin-1, -2, and -3 were not [48]. In analogy, we would propose that claudin-19 could be part of a lipid raft (TAL) or not (CD) depending on the structure and composition of the respective TJ.

Increasing osmolalities in the cultivation medium dramatically decreased R_te_ and increased ion permeability, especially for Cl^−^. High osmolalities on both sides of the IMCD epithelium mimic conditions of strong antidiuresis, also independent of the original stimulus by AVP [49]. The gradual removal of claudin-19 into intracellular compartments and vesicular structures (Figure 8) could partially explain the reduction in R_te_. However, literature and our own first findings emphasize the complexity of the regulation and composition of the paracellular pathway. A parallel increase in claudin-4 as described by Lanaspa et al. [39] could contribute in addition, especially with regard to the increase in anion selectivity. Claudin-8, a direct interaction partner of claudin-4, however, does not show regulation, at least on the level of subcellular localization (Figure 9). Under high osmotic conditions on both sides of the epithelium, osmotic pressure across the epithelium is low and, potentially, tricellular sealing, an additional route for water, ions, and macromolecules, could be less stringent [50]. One indicator for this could be the slightly increasing permeability for the high-molecular 40 kD FITC dextran marker in 600-IMCD and especially 900-IMCD. Other claudins (e.g., claudin-7) and expressional or post-translational regulations of TJ properties have to be considered in future studies to finally complete the picture.

Although AVP is the main controlling hormone of renal water handling [4,49], we could corroborate that local osmolality changes were able to regulate IMCD transport properties and claudin expression independently, similar to the findings of Lanaspa and coworkers [38,39]. Whereas in these studies TJ proteins have been described as targets for regulation, intriguingly, it has also been proposed that the TJ itself could serve as sensor for osmolality and tonicity changes, thereby becoming the origin of regulation (reviewed in [51]). In MDCK cells, acute changes in transepithelial osmotic gradients dramatically changed paracellular selectivity and claudin localization as well as cytoskeleton remodeling [52]. Besides these changes by cell culture osmolality, AVP itself showed effects on paracellular properties as it increased cation selectivity in 300-IMCD. However, these changes were independent of claudin-19, or at least not dependent on subcellular localization of the claudin. We speculate from findings in medullary TAL that mechanisms like claudin-19 phosphorylation could be part of the effect of AVP. In TAL, we could show that AVP directly increased cation selectivity within minutes, without changing claudin-10b localization, indicating that phosphorylation rather than recruitment to the membrane may be involved [53].

In conclusion, we propose that claudin-19 is part of the collecting duct TJ complex. Its presence in the TJ is under the control of ambient interstitial tonicity. Under iso-osmotic conditions, it is responsible for a high electrical transepithelial resistance and contributes to the tonicity-induced changes in paracellular ion selectivity.

## 4. Materials and Methods

### 4.1. Animals

All experiments were performed in accordance with the German law on animal protection and approved by the local authorities (animal ethics protocol number V312-72241.121-2). C57 Bl6/J mice, Wistar or Sprague-Dawley rats were housed under a 12 h light cycle with free access to water and chow.

### 4.2. Cell Culture

Cell culture medium of 300 mosm/kg was used as basic medium. It consisted of high glucose DMEM (PAA Laboratories, Coelbe, Germany) enriched with 1% Ultroser G (CytoGen, Wetzlar, Germany), 1% nonessential amino acids (PAA Laboratories), 1% L-Glutamine (PAA Laboratories; 200 mmol/L), and 1% penicillin/streptomycin (PAA Laboratories). For 600 mosm/kg medium, 100 mmol/L NaCl and 100 mmol/L urea were added; for 900 mosm/kg medium, 200 mmol/L NaCl and 200 mmol/L urea were added, respectively.

IMCD cells for primary cell cultures were isolated as described previously [25,26]. Briefly, 200–300 g Wistar or Sprague-Dawley rats (*n* = 20) of both sexes were sacrificed. Under sterile conditions, the inner medulla of both kidneys was dissected, transferred to enzyme solution (0.2% hyaluronidase type I (Sigma Aldrich, St. Louis, MO, USA) and 0.2% collagenase type II (Biochrom AG, Berlin, Germany) in PBS and digested for 90 min in a thermoshaker (37 °C, 850 rpm). The suspension of cells and tubular fragments was washed in PBS twice by centrifugation und resuspension in 600 mosm/kg medium. Cells were seeded on collagen IV-coated (mouse collagen IV, BD Biosciences, Heidleberg, Germany; 0.2 µg/cm) Costar Transwell Permeable supports (Sigma Aldrich, St. Louis, USA); 0.33 cm^2^) at densities of 350,000 cells/cm^2^ or 600,000 cells/cm^2^. Cells were cultivated at 37 °C and 8% CO_2_. After a 24 h settlement period in 600 mosm/kg medium, cells were transferred to 300, 600, or 900 mosm/kg medium, respectively, and cultivation lasted 5–7 days in total. Medium was changed and transepithelial resistance was monitored daily (EVOM2µ, World Precision Instruments). All filters were checked for confluency after the experimental procedure by visual control for complete epithelial coverage of the filter. Filters with gaps in confluency were excluded from analysis.

### 4.3. Immunohistochemistry

After Ussing chamber measurements, the filters were fixed in 0.4% PFA in PBS at 4 °C overnight. In addition, cryosections (3 µm) of perfusion fixed kidneys of Sprague-Dawley rats and of C57Bl/6 J mice [54] were heated in 0.3% triton-x-100 in PBS (PBST-T) for antigen retrieval. Filters as well as kidney slices were washed extensively in PBS-T and blocked with 1% bovine serum albumin in PBS-T. Filters and cryosections were incubated with primary antibody in 1% BSA overnight at 4°C. The claudin-19 antibody, a kind gift of J. Hou (Department of Internal Medicine, Washington University Renal Division, USA), has been successfully used in a Claudin-19 KD animal model [21]) and was used in a dilution of 1:300, Antibodies used in addition: rabbit anti-aquaporin-, 1:100 (Alomone labs, Jerusalem, Israel), rabbit anti-claudin-8 and rabbit anti-ZO-1 (both Thermo Fisher, Waltham, USA), guinea pig anti-AE1 (kind gift of C. Wagner, University of Zürich, Institute of Physiology, Zürich, Switzerland). After three additional washing steps (PBS-T), filters were incubated with the respective secondary antibody (1:400; AlexaFluo 488, donkey anti-rabbit, AlexaFluo 633, goat anti-rabbit; AlexaFluo 488 goat anti-guinea pig, Thermo Fisher) for 60 min at room temperature. After final extensive washing (PBS-T), filters and cryosections were embedded in Moviol-Dabco. Pictures were taken either confocally (LSM 510, Axiovert 200 M; Zeiss, Jena, Germany) or with Apotome2 (AX 10, Zeiss). Filters were analyzed for the subcellular staining pattern of claudin-19 in three categories as ordinal scale reflecting the functional expression of claudin-10 (see scheme in Figure 8: score 3 for membrane associated tight junction staining (clear lines), score 2 for submembrane staining (diffuse scattering lines), and score 1 for vesicular staining (dotted intracellular staining). For each filter the percentage of the different categories was given with 5–10% precision and each filter was attributed then an overall TJ score between 1 (all claudin-19 in intracellular vesicles) and 3 (all claudin-19 in TJ).

### 4.4. Western Blotting

Enzymatic and enzymatically assisted dissection was used to generate sorted tubular samples [27,55]. After flank incision under deep anesthesia the left kidney of Sprague-Dawley rats was perfused with enzyme solution: 2 mg/mL collagenase Type II in incubation solution (in mmol/L: 140 NaCl, 0.4 KH_2_PO_4_, 1.6 K_2_HPO_4_, 1 MgSO_4_, 10 sodium acetate, 1 α-ketoglutarate, 1.3 calcium gluconate, 5 glycine, containing 48 mg/L trypsin inhibitor and 25 mg/L DNase I, pH 7.4, at 37 °C). Mice kidneys were similarly perfused via the abdominal vein [45]. Tubular segments from 6–8 week C57 Bl/6J or Sprague-Dawley rats were sorted after digestion as described in [45,55] or dissected manually from thin transversal kidney slices at 4 °C in sorting solution (incubation solution supplemented with 0.5 mg/L albumin). For Western blotting, approximately 20–50 tubules per segment were sorted and stored in 20–25 µL 1× Laemmli solution at −80 °C prior to use. For membrane protein solubilization, rat kidneys (6–8 week) were cut in transversal slices at 4 °C and under visual control the papilla (inner medulla, IM) and outer medulla (OM) were prepared, the later with a safety margin to cortex. Therefore, OM mainly comprises the inner stripe. Tissues were snap frozen in liquid nitrogen and stored at −80 °C. After thawing tissues were homogenized with a glass teflon homogenizer in Triton buffer (in mmol/L: 20 Tris–HCl, 50 NaCl, 2 EDTA, 1% Triton-x-100, pH 7.6, protease inhibitor cocktail (Roche, Basel, Switzerland) and incubated for 2 h on ice. After 30 min centrifugation at 21,000× *g* and 4 °C, the supernatant was transferred to a new reaction tube, a sample was kept for determination of protein content (NanoDrop, Thermo Scientific, Waltham, MA, USA) and the tube stored at −20 °C after addition of Laemmli concentrate. The remaining pellet was resuspended in SDS buffer (in mmol/L: 20 Tris–HCl, 50 NaCl, 2 EDTA, 1% SDS, pH 7.6, protease inhibitor cocktail (Roche)), incubated for one additional hour at 4 °C, and then processed as described for the Triton samples. After thawing and denaturation, samples were separated in 15% polyacrylamide minigels and electrophoretically transferred to nitrocellulose membranes. Membranes were blocked for 60 min with 5% BSA in PBS–Tween (0.1% Tween in PBS) and incubated with primary antibody (anti-claudin-19, 1:2000) at 4 °C overnight. Membranes were further incubated for 2 h at RT with the secondary antibodies (Dianova, goat anti-rabbit-HRP) and imaged using a ChemiDoc Imaging system (Biorad, Herkules, USA). Procedure was repeated with anti-β-actin for loading control.

### 4.5. Electrophysiology

IMCD cells grown on filter supports were measured in a modified Ussing Chamber (EP Devices, Leuven, Belgium). Measurements were performed under the osmolality corresponding to the respective cultivation conditions and experimental solutions are enlisted in Table 1. After a short equilibration period, luminal amiloride (50 µmol/L) was applied to inhibit ENaC driven transepithelial Na^+^ transport. In the presence of luminal amiloride, the transepithelial voltage was close to zero in all cases. Next, diffusion potentials were obtained by consecutively replacing the luminal solution by low-NaCl solution and application of a 1:5 luminal to basolateral NaCl concentration gradient (iso-osmotic; 30, 50, and 70 mmol/L NaCl, respectively, see Table 1). All obtained potentials were corrected for their respective calculated liquid junction potentials [56]. Permeability ratio P_Na_/P_Cl_ was calculated using the Goldman–Hodgkin–Katz flux equation. Absolute permeability of Na^+^ was obtained using the simplified Kimizuka–Koketsu equation [54,57]. Absolute permeability for Cl^−^ was calculated from the respective permeability ratio and P_Na_.

### 4.6. FITC Dextran Permeability Measurement

The 40 kDa FITC–dextran conjugate ((Sigma Aldrich, St. Louis, USA) stock solution was dialyzed (8000–10,000 MWCO) to remove free FITC. Under cell culture conditions, 26 µM FITC dextran was added to the luminal compartment of nine filters per osmolality condition. Fluorescence in the basolateral compartment was measured at time points 0 and 30 min in a plate reader (Tecan, Männedorf, Switzerland). Calibration curves were conducted to calculate FITC–dextran concentration. FITC–dextran flow rate was given as change in concentration over time in the chamber volume. Permeability coefficient (P) was calculated relating the flow rate to the respective filter area and the initial concentration difference as driving force. Permeability of empty filters under the different osmolality conditions was determined accordingly.

### 4.7. Electrolyte Measurement

Luminal and basolateral cell culture supernatants of the last 24 h of cultivation were collected, Na^+^ and K^+^ concentrations measured by flame photometry (EFOX 5053, Eppendorf, Hamburg, Germany), and basolateral to luminal differences in electrolyte concentrations were calculated.

### 4.8. Statistics

Electrophysiological data are presented as means ± SEM. Two groups were tested by unpaired *t*-test or Mann–Whitney test. Three groups were tested with one-way ANOVA followed by Tukey post-hoc testing or Kruskal–Wallis test and Dunn’s post-hoc test. All statistical analyses were performed using GraphPad Prism including the test for normal distribution (D’Agostino & Pearson normality test). Immunofluorescence subcellular staining data are presented as a stacked-column graph, giving the respective category as a percentage. Filter-scores were tested with nonparametric Mann–Whitney *U*-Test or Kruskal–Wallis test and Dunn’s post-hoc test.

## Figures and Tables

**Table 1:** Composition of experimental solutions used in Ussing chamber experiments. Concentrations are given in mmol/L. pH was adjusted to pH 7.4.

	Control Solution			Low NaCl Solution		
	‘300‘	‘600‘	‘900‘	‘300‘	‘600‘	‘900‘
NaCl	145	245	345	30	50	70
KH_2_PO_4_	0.4	0.4	0.4	0.4	0.4	0.4
K_2_HPO_4_	1.6	1.6	1.6	1.6	1.6	1.6
MgCl_2_	1	1	1	1	1	1
Ca-gluconate	1.3	1.3	1.3	1.3	1.3	1.3
glucose	5	5	5	5	5	5
urea	-	100	200	-	100	200
mannitol	-	-	-	230	390	550

## Abbreviations

IMCD	Inner medullary collecting duct
AVP	Arginine vasopressin
PG2	Prostaglandin E2
TJ	Tight junction
TAL	Thick ascending limb
PT	Proximal tubule
PCT	Proximal convoluted tubule
PST	Proximal straight
DCT	Distal convoluted tubule
CNT/CD	Connecting tubule and cortical collecting duct
mCD	Medullary collecting duct (outer stripe of outer medulla)
SDS	sodium dodecyl sulfate
AQP2	Aquaporin 2
V_te_	Transepithelial voltage
R_te_	Transepithelial resistance
I’_sc_	Equivalent short circuit current
TER	Transepithelial resistance (cell culture)
DP	Diffusion potential
